# Novel Cysteine-Centered Sulfur Metabolic Pathway in the Thermotolerant Methylotrophic Yeast *Hansenula polymorpha*

**DOI:** 10.1371/journal.pone.0100725

**Published:** 2014-06-24

**Authors:** Min Jeong Sohn, Su Jin Yoo, Doo-Byoung Oh, Ohsuk Kwon, Sang Yup Lee, Andriy A. Sibirny, Hyun Ah Kang

**Affiliations:** 1 Department of Life Science, Chung-Ang University, Seoul, Korea; 2 Department of Chemical & Biomolecular Engineering (BK21+ program), Korea Advanced Institute of Science and Technology (KAIST), Daejeon, Korea; 3 Biochemicals and Synthetic Biology Research Center, Korea Research Institute of Bioscience and Biotechnology (KRIBB), Daejeon, Korea; 4 Institute of Cell Biology, National Academy of Sciences of Ukraine, Lviv, Ukraine; University of Louisville, United States of America

## Abstract

In yeast and filamentous fungi, sulfide can be condensed either with *O*-acetylhomoserine to generate homocysteine, the precursor of methionine, or with *O*-acetylserine to directly generate cysteine. The resulting homocysteine and cysteine can be interconverted through transsulfuration pathway. Here, we systematically analyzed the sulfur metabolic pathway of the thermotolerant methylotrophic yeast *Hansenula polymorpha*, which has attracted much attention as an industrial yeast strain for various biotechnological applications. Quite interestingly, the detailed sulfur metabolic pathway of *H. polymorpha,* which was reconstructed based on combined analyses of the genome sequences and validation by systematic gene deletion experiments, revealed the absence of *de novo* synthesis of homocysteine from inorganic sulfur in this yeast. Thus, the direct biosynthesis of cysteine from sulfide is the only pathway of synthesizing sulfur amino acids from inorganic sulfur in *H. polymorpha*, despite the presence of both directions of transsulfuration pathway Moreover, only cysteine, but no other sulfur amino acid, was able to repress the expression of a subset of sulfur genes, suggesting its central and exclusive role in the control of *H. polymorpha* sulfur metabolism. ^35^S-Cys was more efficiently incorporated into intracellular sulfur compounds such as glutathione than ^35^S-Met in *H. polymorpha*, further supporting the cysteine-centered sulfur pathway. This is the first report on the novel features of *H. polymorpha* sulfur metabolic pathway, which are noticeably distinct from those of other yeast and filamentous fungal species.

## Introduction

**Figure 1:**
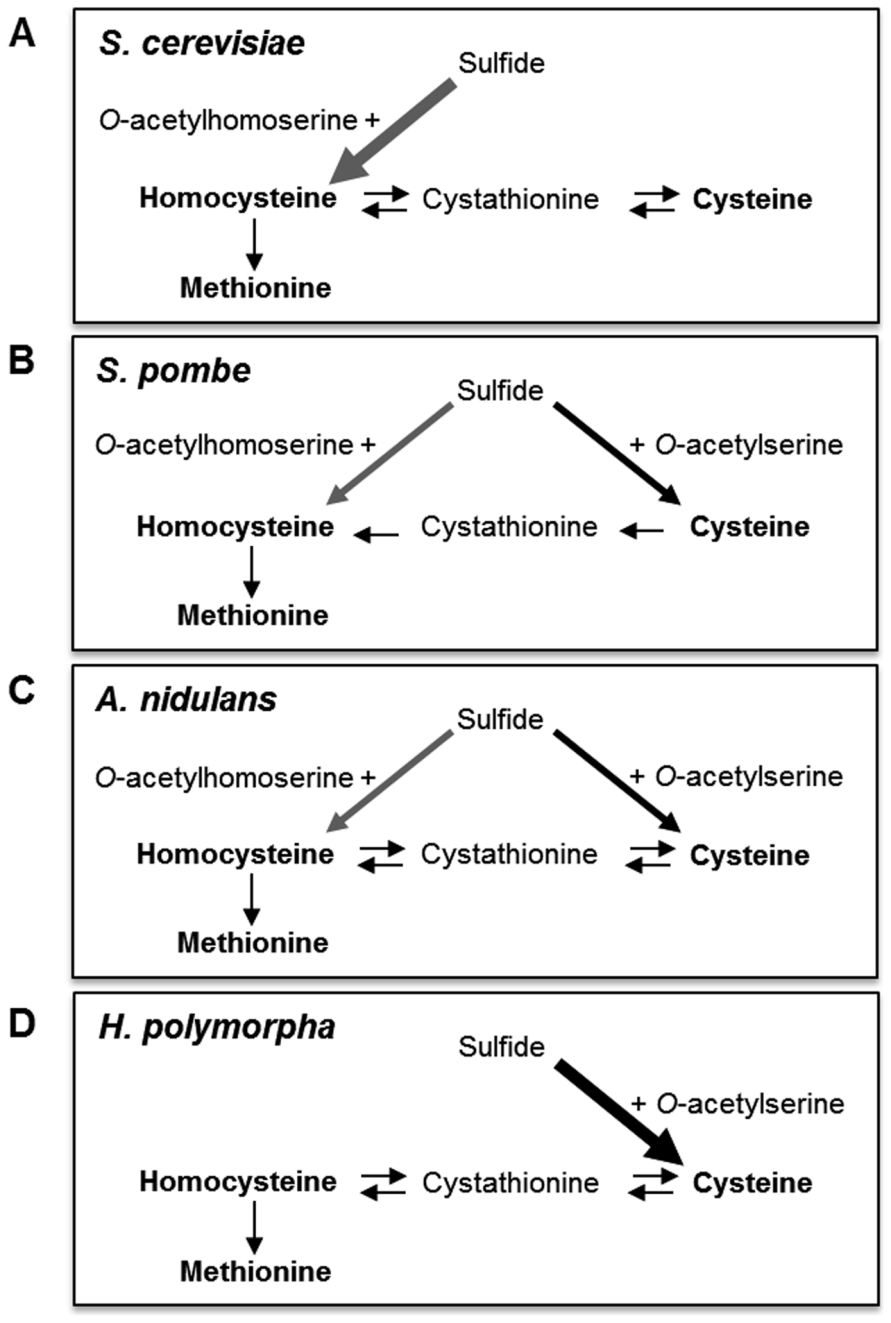
Different pathways of sulfur incorporation into carbon chains and transsulfuration in yeast and filamentous fungal species. Inorganic sulfide can be combined with *O*-acetylhomoserine or *O*-acetylserine to produce homocysteine and cysteine, respectively. The budding yeasts *S. cerevisiae* (A) has only *O*-acetylhomoserin pathway for sulfide incorporation, whereas the fission yeast *S. pombe* (B) and the filamentous fungus *A. nidulans* (C) possess both pathways. The present study proposes that *H. polymorpha* (D) has only *O*-acetylserine pathway.

Sulfur plays important roles in a number of cellular processes, such as the redox cycle (thioredoxins, glutaredoxins), stress response (glutathione, phytochelatins), enzyme reactions (iron-sulfur cluster as prosthetic group), and metabolism of secondary products (glucosinolates, sulfated compounds) [1], [2]. It is also essential in C1 metabolism as a source of reduced sulfur for the biosynthesis of *S*-adenosyl-methionine (AdoMet). Cellular requirements for sulfur can be fulfilled by the uptake of sulfur-containing amino acids, cysteine and methionine, or by the assimilation of inorganic sulfur into organic compounds such as cysteine and homocysteine, which are used for further biosynthesis of glutathione (GSH) and methionine, respectively [3]. Different from microorganisms and plants, animals do not have the assimilatory mechanisms for inorganic sulfur, and they require methionine as an essential amino acid for their source of sulfur nutrient. In the yeast and filamentous fungal species, cysteine biosynthesis from sulfide can be divided into two pathways [4], [5]. In one pathway, sulfide is condensed with *O*-acetylhomoserine to generate homocysteine, which can be converted to cystathionine and then to cysteine, as in *Saccharomyces cerevisiae* (Fig.1A). In the other pathway, sulfide is condensed with *O*-acetylserine to generate cysteine in a process catalyzed by cysteine synthase (OAS pathway), as in *Schizosaccharomyces pombe* (Fig.1B). The filamentous fungi *Aspergillus nidulans* and *Neurospora crassa* employ both pathways for cysteine biosynthesis (Fig. 1C). In *S. cerevisiae*, *A. nidulans,* and *N. crassa,* cysteine and homocysteine interconvert through forward and reverse transsulfuration pathways. In contrast, *S. pombe* lacks the reverse pathway for conversion of homocysteine to cysteine due to lack of required enzymes cystathionine β-synthase and cystathionine γ-lyase [6], [7].

The thermotolerant methylotrophic yeast *Hansenula polymorpha* is characterized by its high tolerance to various stresses induced by heavy metals, xenobiotics (drugs), and environmental pollutants. As a consequence it has attracted much attention as a promising host strain for recombinant protein production [8] but also as an industrial yeast strain for various biotechnological applications [9]. In basic research, it has long been used as a favorable model system to study peroxisome biogenesis and function due to extensive peroxisome proliferation during growth on methanol [10]. Since methylotrophic yeasts are obligatorily dependent on thiol GSH for oxidation and detoxification of formaldehyde, a toxic methanol oxidation intermediate [11], *H. polymorpha* is considered as a good model system to study the metabolism and functions of GSH [12], [13] and a promising host strain for high level production of GSH [14]. Even though the sulfur metabolism is important for methylotrophic growth of *H. polymorpha*, its metabolic pathways involved in the biosynthesis of GSH and other sulfur compounds is not well understood.

In this study, a detailed sulfur metabolic pathway of *H. polymorpha* was reconstructed based on combined analyses of the genome sequences and validation by systematic gene deletion experiments. In addition, we examined the effect of sulfur-containing amino acids on the transcriptional regulation of *H. polymorpha* sulfur pathway. Here, we showed the novel features of the sulfur metabolic pathway and its regulation in *H. polymorpha*, which are notably distinct from those of other yeast and filamentous fungal species.

## Materials and Methods

### Strains, media and cultivation conditions

The *H. polymorph* DL1-LdU (*leu2 ura3::lac*Z) strain was used as a parental strain and the mutant *H. polymorpha* strains constructed in this study are listed in Table 1. The null mutant strain of *ScGSH1* was constructed by replacing the coding region of *ScGSH1* with the *ScLEU2* gene in *S. cerevisiae* L3262 (*MATα ura3–52 leu2–3, 112 his4–34*). Yeast cells were cultivated in YPD (1% yeast extract, 2% peptone, and 2% glucose), YNB (0.67% yeast nitrogen base without amino acids and 2% glucose) or sulfur-free B-medium (synthetic medium with 2% glucose without any sulfur source) [4]. Inorganic (sulfate, sulfite, and sulfide) and organic (methionine, cysteine, cystathionine, homocysteine, and GSH) sulfur sources were added to B-medium at 2 mM and 0.2 mM final concentration, respectively. For solid B-medium, 1% agarose was used instead of agar to minimize the addition of uncontrolled sulfur sources to the medium. Uracil (20 µg/ml), leucine (100 µg/ml), and histidine (20 µg/ml) were added to the growth media according to the auxotrophic requirements of individual strains. For heavy metal cadmium exposure, yeast cells were cultured in YPD containing cadmium sulfate (C2919, Sigma-Aldrich, St. Louis, MO, USA).

**Table 1:** Yeast strains used in this study.

Strain	Genotype	Reference
*H. polymorpha*		
DL1-L	*leu2*	Kang *et al*., 2002
DL1-LdU	*leu2 ura3::lacZ*	Kang *et al*., 2002
*Hpgsh1*Δ	*leu2 ura3::lacZ trp1::lacZ gsh1::TRP1*	Cheon *et al*., 2009
*Hpcys1*Δ	*leu2 ura3::lacZ, cys1::URA3*	This study
*Hpcys1*Δ*Hpcys4abc*Δ	*leu2 ura3::lacZ, cys1::lacZ, cys4a::lacZ, cys4b::LEU2 cys4c::URA3*	This study
*Hpcys1*Δ*Hpcys3*Δ	*leu2 ura3::lacZ cys1::lacZ, cys3::URA3*	This study
*Hpcys1*Δ*Hpstr3*Δ	*leu2 ura3::lacZ cys1::lacZ, str3::URA3*	This study
*Hpcys1*Δ*Hpstr2*Δ	*leu2 ura3::lacZ cys1::lacZ, str2::URA3*	This study
*Hpmet3*Δ	*leu2 ura3::lacZ met3::URA3*	This study
*Hpmet2*Δ	*leu2 ura3::lacZ met2::URA3*	Cheon *et al.*, 2009
*Hpsat1*Δ	*leu2 ura3::lacZ sat1::URA3*	This study
*S. cerevisiae*		
L3262	*MATα ura3–52 leu2–3, 112 his4–34*	Kang *et al*., 1998
*Scgsh1*Δ	*MATα ura3–52 leu2–3, 112 his4–34 gsh1::ScLEU2*	This study

### Construction of mutant strains deficient in OAS pathway and transsulfuration pathway

The complete DNA sequences of genes involved in cysteine and methionine biosynthesis were obtained from the in-house database of *H. polymorpha* DL1-L (Korea Research Institute of Bioscience and Biotechnology, Korea). Disruption of these genes was carried out by the modified fusion PCR-based gene deletion method as described previously [15] using gene-specific primers (Table S1). The *H. polymorpha* DL1-LdU (*leu2 ura3*) strain was transformed with PCR product carrying the gene disruption cassette with the *HpURA3* pop-out marker and transformants were selected on SC-URA medium supplemented with 0.1 mM GSH, methionine, or cysteine. Correct replacement of the target gene was confirmed by PCR analysis using primers NF and CR. To generate multiple gene disruptions, *ura3* revertants of a disruption mutant were selected on YPD agar plates containing 5-fluoroorotic acid (5-FOA; 0.5 mg/ml) and subjected to the next round of the fusion PCR-based gene deletion method.

### Quantitative reverse transcription PCR analysis

For quantitative reverse transcription PCR (qRT-PCR) analysis, cDNA was synthesized from total RNA using Superscript reverse transcriptase (Invitrogen, Carlsbad, CA). qRT-PCR was performed in Rotor-Gene Q (Qiagen) with a QuantiMix SYBR Kit (Philekorea Technology, Daejeon, Korea) using gene-specific primer sets (Table S1). Each sample was analyzed in duplicate and normalized to β-actin as an endogenous control. The relative concentrations of mRNAs were calculated using 2^−ΔΔCt^ methodology.

### Analysis of GSH synthesis by ^35^S labeling

Yeast cells were cultivated in YNB medium supplemented with uracil, leucine, and histidine at 30°C for *S. cerevisiae* and 37°C for *H. polymorpha.* When the yeast culture reached mid-log phase (*A*_600_ = 0.3), 2-ml aliquots were withdrawn and incubated with [^35^S]methionine (200 mCi) or [^35^S]cysteine (200 mCi) for 2 hr in the presence of 0, 0.6, or 2 mM CdSO_4_. Cells were collected by centrifugation, washed with water, and resuspended in 50 µl of water. Cells were then boiled for 5 min and centrifuged to obtain the ^35^S-labeled metabolites in the supernatant. The metabolites were oxidized by addition of an equal volume of performic acid [16], which converts both oxidized and reduced forms of glutathione to glutathione-sulfonic acid. Samples (5 µl) were applied onto cellulose thin layer chromatography in the following solvent system: butanol-1/acetic acid/water (90∶15∶33) [17]. The ^35^S-labeled metabolites on the thin layer chromatography plate were scanned and quantified by phosphor technology (BAS-1500, FUSIFILM, Japan).

### Bioinformatic analysis

Multiple sequence alignments were constructed with CLUSTALW method of the DNASTAR MegAlign program and shaded using GeneDoc (http://www.nrbsc.org). Percentage identity was calculated by comparing sequence pairs in relation to the phylogeny reconstructed by the CLUSTALW method of the DNASTAR MegAlign program [18].

## Results

### *In silico* reconstruction of the sulfur metabolic pathway in *H. polymorpha*

**Figure 2:**
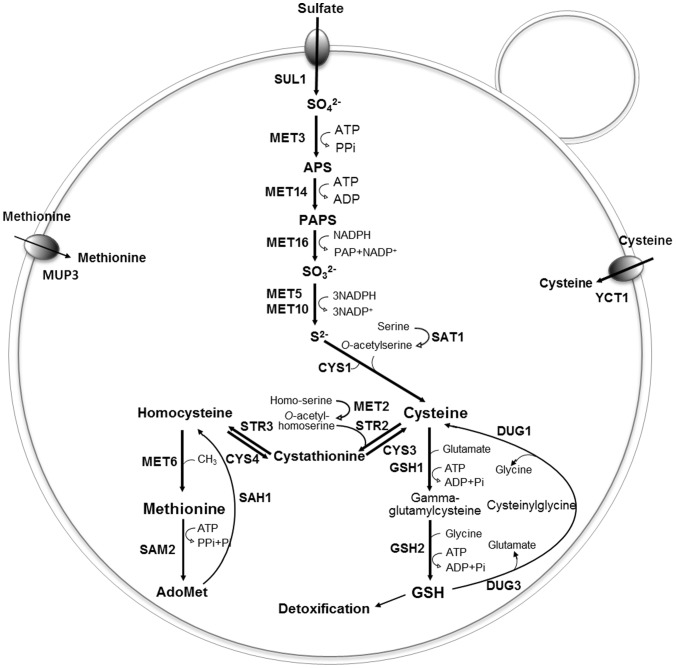
Schematic representation of a reconstructed sulfur pathway of *H. polymorpha*. The putative sulfur metabolism pathway involved in sulfate assimilation, sulfur amino acid biosynthesis, and the methionine salvage pathway was constructed based on in-house genome information on *H. polymorpha* DL-1 strain (KRIBB in Korea). The nucleotide sequences of other *H. polymorpha* genes involved in sulfur assimilation and sulfur amino acid biosynthetic pathway were deposited under accession numbers JN676924-JN676946 (Table S2).

**Figure 3:**
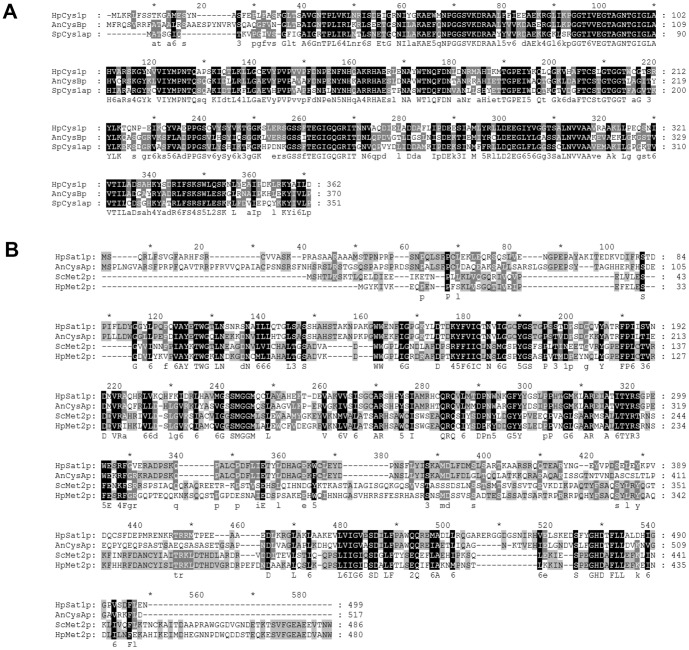
Sequence alignment of HpCys1p (A) and HpSat1p (B) with other yeast and filamentous fungal homologs. AnCysBp, *A. nidulans cysB* protein; SpCys1ap, *S. pombe CYSla* protein. AnCysAp, *A. nidulans cysA* protein; ScMet2p, *S. cerevisiae MET2* protein; HpMet2p, *H. polymorpha MET2* protein.

Based on in-house genome information on the *H. polymorpha* DL-1 strain (KRIBB in Korea), we have reconstructed the putative sulfur metabolism pathway involved in sulfate assimilation, sulfur amino acid biosynthesis, and the methionine salvage pathway (Fig. 2). The nucleotide sequences of *H. polymorpha* DL-1 genes involved in sulfur metabolic pathway were deposited in GenBank under accession numbers JN676924-JN676946 (Table S2). For sulfur incorporation into carbon chains, we identified a *H. polymorpha* gene predicted to participate in the direct biosynthesis of cysteine from inorganic sulfur compounds, namely the cysteine synthase gene, which is absent in *S. cerevisiae* and *Candida glabrata* [19]. Using the *A. nidulans cysB* and *S. pombe CYS1a* genes encoding cysteine synthase as queries for the search, we identified a candidate ORF designated *HpCYS1* whose deduced amino acid sequence shows 59% and 54% identity to those of *A. nidulans cysB* and *S. pombe CYS1a* proteins, respectively (Fig. 3A). The putative *HpCYS1* protein (HpCys1p) has two lysine residues that can bind the pyridoxal 5′-phosphate cofactor and are highly conserved among cysteine synthases of plants and bacteria. The presence of a functional OAS pathway in *H. polymorpha* was further suggested by the identification of a *H. polymorpha* homolog (designated *HpSAT1*) of *A. nidulans cysA* encoding serine *O*-acetyltransferase [20], which could provide a substrate of HpCys1p. The putative *HpSAT1* protein (HpSat1p) showed high identity to *A. nidulans* CysAp (59.0%) compared to low identities to ScMet2p (24.1%) and HpMet2p (27.3%) encoding homoserine *O*-acetyltransferase (Fig. 3B).

Interestingly, analysis of the *H. polymorpha* genome indicates that *H. polymorpha* does not have an ORF coding for *O*-acetylhomoserine sulfhydrylase or homocysteine synthase (encoded by *MET17* or *MET25* in *S. cerevisiae*), which catalyzes the synthesis of homocysteine from homoserine by incorporation of sulfide. In fact, the absence of a *MET17* homolog in *H. poymorpha* was implicated in a previous study reporting the methionine auxotrophic phenotype of a *H. poymorpha* mutant strain with a defect in a gene coding for a homolog of *S. cerevisiae* Str3p [21]. Considering that homocysteine is the central molecule for the biosynthesis of sulfur amino acids in all organisms studied [19], the absence of *de novo* synthesis of homocysteine from inorganic sulfur might be an exceptional feature of *H. polymorpha*. We also searched for other *H. polymorpha* genes showing significant identities to *S. cerevisiae* genes involved in the transsulfuration pathway, including *HpSTR2* (cystathionine *γ-*synthase) and *HpSTR3* (cystathionine *β-*lyase) for forward transsulfuration, and *HpCYS3* (cystathionine *γ-*lyase) for reverse transsulfuration. Three *H. polymorpha* ORFs with quite low identity to *ScCYS4* (cystathionine *β*-synthase) were identified and designated *HpCYS4a*, *HpCYS4b,* and *HpCYS4c*. For the methyl cycle of methionine and AdoMet biosynthesis, only one copy of a *SAM2* homolog was identified in the *H. polymorpha* genome, whereas two copies of genes encoding *S*-adenosylmethionine synthetase are present in *S. cerevisiae* (Table S2).

### Validation of *H. polymorpha* sulfur pathway by systematic deletion analysis

**Figure 4:**
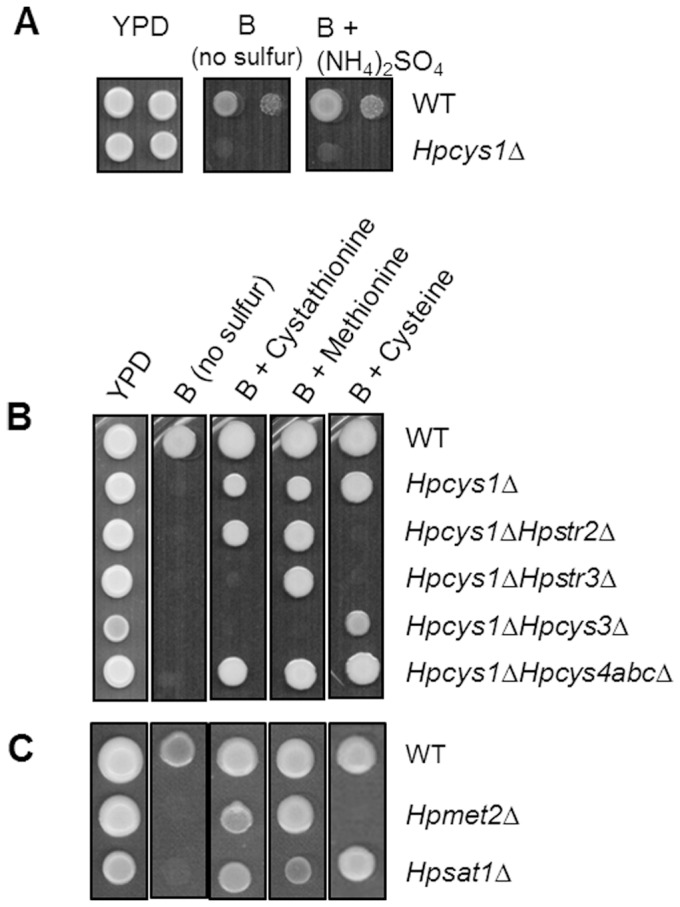
Validation of the presence of the complete transsulfuration pathway by gene deletion analysis. A set of *H. polymorpha* mutant strains in the sulfur assimilation and transsulfuration pathway were analyzed for their growth on B-medium supplemented with various sulfur sources. (A) Growth analysis of *Hpcys1*Δ on B-medium with inorganic sulfur source, NH_4_SO_4_. (B) Growth analysis of *Hpcys1*Δ, *Hpcys1*Δ*Hpstr2*Δ, *Hpcys1*Δ*Hpstr3*Δ, *Hpcys1*Δ*Hpcys3*Δ, and *Hpcys1*Δ*Hpcys4abc*Δ on B medium with organic sulfur compounds as sole sulfur source. (C) Growth analysis of *Hpmet2*Δ and *Hpsat1*Δ on B-medium with organic sulfur compounds as sole sulfur source.

To determine whether homologs identified by *in silico* analysis play physiological roles in sulfur metabolism, a set of *H. polymorpha* mutants were constructed by gene deletion (Table 1). Phenotypic analysis of *H. polymorpha* mutant strains was performed on B-media supplied with sulfate, cystathionine, cysteine, or methionine as the only sulfur source. Since *H. polymorpha* does not possess a *MET17* homolog, the reaction mediated by HpCys1p should be the only way to synthesize the sulfur amino acid *de novo* from inorganic sulfur. As predicted by the absence of *MET17* in *H. polymorpha,* the *Hpcys1*Δ mutant strain was not able to grow on B medium supplemented with sulfate as the only sulfur source (Fig. 4A). Growth of *Hpcys1*Δ on B-medium was recovered by supplementation with any of cystathionine, cysteine, or methionine as a sole sulfur source (Fig. 4B), strongly indicating that cysteine and methionine can be interconverted in *H. polymorpha* through the transsulfuration pathway in both directions with cystathionine as an intermediate.

The transsulfuration reactions involve at least four enzymes, Str2p, Str3p, Cys3p, and Cys4p [5], [7], [22], [23]. To more systematically validate the presence of an active transsulfuration pathway in both directions with allocation of functional genes in *H. polymorpha,* a set of double disruption mutants (*Hpcys1*Δ*cys3*Δ, *Hpcys1*Δ*str2*Δ, *Hpcys1*Δ*str3*Δ, *Hpcys1*Δ*cys4a*Δ, and *Hpcys1*Δ*cys4b*Δ) and a quadruple disruptant (*Hpcys1*Δ*cys4a*Δ*cys4b*Δ*cys4c*Δ) were constructed in the background of the *Hpcys1*Δ strain. Although all deletion mutants were viable and morphologically normal in YPD medium, they displayed growth defects to different degrees on various sulfur sources (Fig. 4B). Growth of the *HpSTR* disruption mutants, such as *Hpcys1*Δ*str2*Δ and *Hpcys1*Δ*str3*Δ, was possible only with supplementation of methionine, but not cysteine, supporting the notion that HpStr2p and HpStr3p are responsible for the forward transsulfuration reaction from cysteine to methionine in *H. polymorpha*. On the other hand, *Hpcys1*Δ*cys3*Δ could only grow on cysteine, but not on cystathionine or methionine (Fig. 4B), strongly supporting the essential role of HpCys3p in the reverse transsulfuration in *H. polymorpha*. Unexpectedly, none of the single deletion strains of *HpCYS4* homologs showed any apparent growth defect phenotype. Even the quadruple *Hpcys1*Δ*cys4a*Δ*cys4b*Δ*cys4c*Δ null mutant was viable and morphologically normal in methionine-supplemented minimal medium (Fig. 4B), indicating that other proteins can perform the function of cystathionine β-synthase.

The presence of an active OAS pathway in *H. polymorpha* was further supported by analysis of the growth phenotypes of the *HpSAT1* disruptant (*Hpsat1*Δ), which were clearly distinctive from those of the *HpMET2* disruptant (*Hpmet2*Δ) with deletion of the *HpMET2* gene encoding homoserine *O*-acetyltransferase [24]. While growth of *Hpsat1*Δ on B-medium can be supported by supplementation with cysteine or methionine, growth of *Hpmet2*Δ is strictly dependent on the addition of methionine (Fig. 4C). The similar growth patterns of *Hpsat1*Δ and *Hpcys1*Δ, as shown in Fig. 4, strongly suggest that the role of HpSat1p is to generate a substrate for the reaction mediated by HpCys1p. In particular, the *Hpcys1*Δ and *Hpsat1*Δ strains showed an apparently reduced growth phenotype compared with the wild type strain even on YPD medium, indicating the importance of the OAS pathway in the generation of cysteine, which serves as the base for the biosynthesis of sulfur amino acids in *H. polymorpha*.

**Figure 5:**
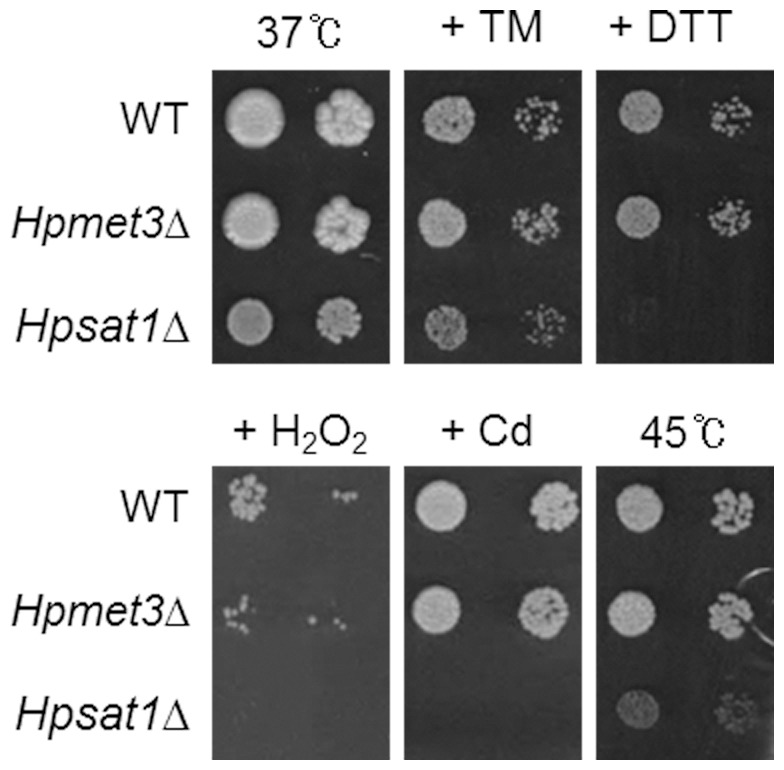
Increased sensitivity of *Hpsat1*Δ mutant strain to Cd, oxidative, and heat stresses. *H. polymorpha* wild-type (WT), *Hpmet3*Δ, and *Hpsat1*Δ strains were spotted on YPD medium containing 0.75 µg/ml tunicamycin (TM), 1 mM CdSO_4_ (Cd), or 30 mM DTT and cultivated at 37°C. For heat stress, yeast cells were cultivated at 45°C.

In methylotrophic yeasts, antioxidant activity is particularly essential for methanol metabolism, which produces hydrogen peroxide [25]. Considering that cysteine is the rate-limiting nutrient in the biosynthesis of GSH, an essential antioxidant molecule involved in oxidative stress response and detoxification for methylotrophic growth, it is plausible that *H. polymorpha* might have evolved to have a predominantly cysteine-centered sulfur pathway to maximize GSH biosynthesis. It is noteworthy that the *Hpsat1*Δ strain displayed increased sensitivity not only to oxidative stress and high temperature but also to Cd (Fig. 5), indicating that *de novo* cysteine biosynthesis from inorganic sulfur via the OAS pathway is an essential step in providing sulfur compounds required for the inherent resistance of *H. polymorpha* to Cd, oxidative, and heat stresses.

### Regulation of the sulfur metabolic pathway in *H. polymorpha* by sulfur amino acids

To obtain information on transcriptional regulation of the sulfur pathway, we performed preliminary northern blot analysis on a few selected genes, such as *HpMET3, HpMET10, HpCYS1,* and *HpCYS3* involved in the sulfur pathway and analyzed the effect of sulfur limitation and Cd exposure on their expression (Figure S1). It was shown that although the transcripts of *HpMET3*, *HpMET10*, and *HpCYS3* were barely detectable in YPD medium, they were highly expressed in medium B, indicating the induced expression under sulfur limitation condition (Figure S1A, left panel). Interestingly, the basal level of the *HpCYS1* transcript was detected significantly high even on YPD medium. Upon exposure to Cd, the induced expression of *HpMET3*, *HpMET10*, and *HpCYS3* was also observed (Figure S1A, right panel). It is notable that the induction of *HpMET3*, *HpMET10*, and *HpCYS3* by sulfur limitation could be repressed significantly by addition of 1 mM cysteine to B-medium, but was not greatly repressed by 1 mM methionine (Figure S1B). Moreover, supplementation with methionine rather strongly induced transcription of *HpCYS1*. Our northern blot data led to speculation that the expression of sulfur metabolic pathway genes in *H. polymorpha* is tightly subject to down-regulation by cysteine, but not by methionine supplementation.

**Figure 6:**
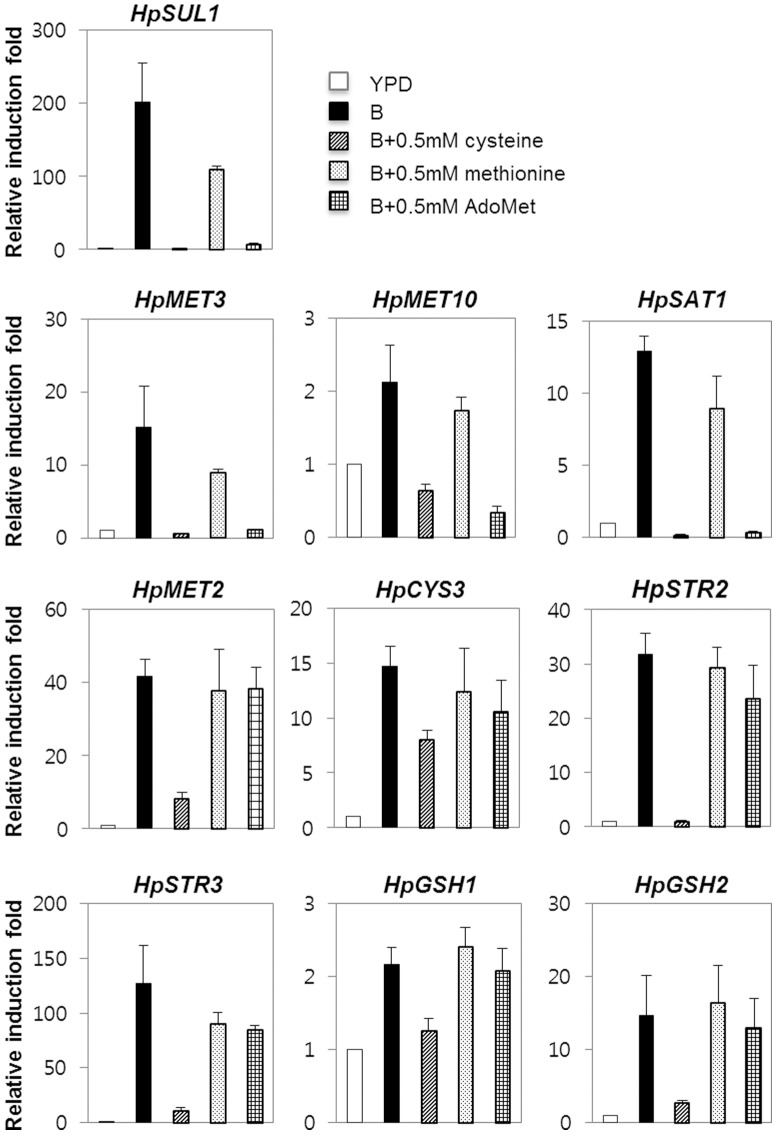
Quantitative real-time PCR analysis of transcriptional regulation of *H. polymorpha* genes in the sulfur pathway. Expression of a selected set of genes was analyzed by qRT-PCR. *H. polymorpha* cells were grown in YPD to the exponential phase and then transferred to B-medium supplemented with the indicated sulfur compounds. After 2 hr cultivation, yeast cells were harvested and total RNA was extracted for analysis. The transcript levels of all genes were corrected according to actin levels, and the induced expression levels are shown as relative ratios to the levels of wild-type cultivated in YPD, respectively. Error bars represent standard deviation of triplicate measurements.

In *S. cerevisiae*, the repression effect of methionine on the expression of sulfur metabolic pathway genes is attributed to its conversion to cysteine [26]. To investigate the possibility that the marginal repressive effect of methionine on the expression of sulfur metabolic pathway genes is due to the intrinsic low uptake rate of methionine by *H. polymorpha*, we examined the repressive effect of supplementation with AdoMet, which would be converted to homocysteine through the methyl cycle and then transformed into cysteine via the reverse transsulfuration pathway (Fig. 6). The quantitative reverse transcription PCR (qRT-PCR) analysis of *HpMET2*, *HpSAT1*, *HpSTR2, HpSTR3*, *HpGSH2*, and *HpCYS3* were carried out, together with *HpSUL1*, *HpMET3*, and *HpMET10*. The RNA samples were obtained from cells cultivated in YPD, B-medium, and B-medium supplemented with 0.5 mM methionine, cysteine, or AdoMet. It was observed that all the tested genes were induced in B-medium as indicated in northern blot analysis. Quite unexpectedly, we found that expression of *HpMET2*, *HpSTR3*, and *HpCYS3*, which are involved in the transsulfuration pathway, was not repressed even by the addition of AdoMet, while the expression of *HpMET3*, *HpSUL1*, and *HpSAT1*, which are involved in sulfur assimilation, was efficiently repressed by the addition of AdoMet (Fig. 6). Semi- quantitative PCR analysis also confirmed the differential effect on transcriptional repression of *H. polymorpha* sulfur genes by methionine, cysteine, and AdoMet (Figure S2). This is an unexpected feature of *H. polymorpha* sulfur metabolism, considering that methionine, AdoMet, and cysteine repress *MET* gene expression equally efficiently in *S. cerevisiae* [26]. We also analyzed the expression pattern of *HpGSH1* and *HpGSH2*, encoding key enzymes responsible for the synthesis of GSH and observed that their expressions were also repressed only by the supplementation of cysteine, not by Met and AdoMet.

### Analysis of glutathione biosynthesis in *H. polymorpha*

**Figure 7:**
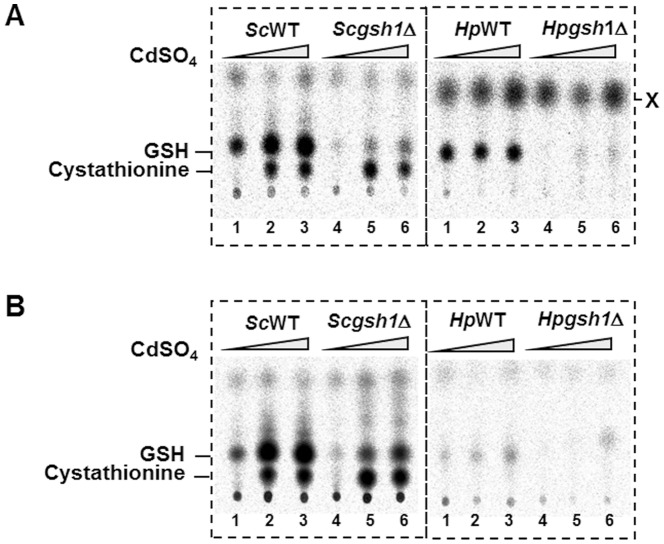
Analysis of GSH biosynthesis by ^35^S-Met and ^35^S-Cys labeling. *S. cerevisiae* (left panel) and *H. polymorpha* (right panel) cells were labeled with ^35^S-Cys (A) or ^35^S-Met (B) during 2 hr incubation in the presence of Cd at the concentration of 0 mM (lanes 1 and 4), 0.6 mM (lanes 2 and 5), and 2 mM (lanes 3 and 6). Intracellular ^35^S-labeled metabolites were extracted and separated by TLC. WT, wild-type strain; *gsh1*Δ, *GSH1* null mutant strain. Some ^35^S-labeled metabolites (GSH, cystathionine, X) are indicated.

Given the importance of sulfur metabolic pathway in the biosynthesis of GSH, which is an essential sulfur compound for detoxification of Cd, we investigate GSH biosynthesis in *H. polymorpha* with comparison to that of *S. cerevisiae* under Cd stress conditions (Fig. 7). After 2 hr incubation with [^35^S]methionine (^35^S-Met) or [^35^S]cysteine (^35^S-Cys) in the presence of 0, 0.6, or 2 mM CdSO_4_, cells were harvested and the extracted metabolites were separated by thin layer chromatography (TLC). Upon Cd treatment, the synthesis level of ^35^S-GSH increased more than 4-fold in the *S. cerevisiae* wild-type strain labeled with either ^35^S-Cys (Fig. 7A, left panel) or ^35^S-Met (Fig. 7B, left panel). In contrast, the ^35^S-GSH biosynthesis from ^35^S-Cys or ^35^S-Met was significantly decreased in *Scgsh1*Δ, as expected. It is interesting to note that the level of ^35^S-cystathionine, an intermediate metabolite in the transsulfuration pathway also increased with an increase in Cd concentration in both labeling reactions. This is consistent with a previous report of an increased flux toward the transsulfuration branch followed by GSH synthesis in *S. cerevisiae* upon exposure to Cd [27]. The residual GSH biosynthesis activity in the *Scgsh1*Δ mutants could be explained by the presence of another minor system for GSH biosynthesis through the Pro1 and Pro2 enzymes involved in proline biosynthesis in *S. cerevisiae* [28].

In the case of *H. polymorpha*, it is quite noteworthy that ^35^S-GSH was detected after 2 hr incubation with ^35^S-Cys (Fig. 7A, right panel) but hardly with ^35^S-Met (Fig. 7B, right panel). Even with ^35^S-Cys, ^35^S-labeled cystathionine was not detected in TLC with the samples from *H. polymorpha*. Moreover, the increase in GSH biosynthesis upon Cd exposure was not as obvious in *H. polymorpha* as in *S. cerevisiae*, although we noticed increased accumulation of an unidentified ^35^S-labeled intermediate, designated X, in a Cd concentration-dependent manner in *H. polymorpha*. In addition, analysis of the total amount of intracellular ^35^S-labeled metabolites showed apparently higher incorporation efficiency of ^35^S-Cys than that of ^35^S-Met in *H. polymorpha* (Figure S3), suggesting intrinsic low uptake capacity of *H. polymorpha* for methionine.

## Discussion

The comparative genomic study of sulfur metabolism of hemiascomycetous yeasts [19] indicated that homocysteine is the base for the biosynthesis of sulfur amino acids in the traditional yeast *S. cerevisiae* and most hemiascomycetous yeasts (Fig. 1A). In *S. cerevisiae*, sulfide is incorporated into a three-carbon chain through formation of homocysteine, followed by synthesis of methionine and cysteine from homocysteine through either the methyl cycle (leading to the synthesis of methionine and AdoMet) or the reverse transsulfuration pathway (leading to synthesis of cysteine and glutathione) [29]. The filamentous fungi, such as *A. nidulans* and *N. crassa*, and the fission yeast have an additional mechanism for sulfide incorporation into carbon chains to give cysteine through the OAS pathway (Fig. 1B and 1C). Moreover, interconversion of sulfur amino acids (methionine-homocysteine-cysteine) occurs through both forward and reverse transsulfuration pathways in the filamentous fungi, which have been reported to have the richest repertoire of sulfur metabolic options. In contrast, *S. pombe* has only one pathway with forward transsulfuration. It was suggested that wild type *S. pombe* can utilize methionine as a sulfur source only after it is degraded to give rise to sulfate [6]. Similar to *S. cerevisiae* and filamentous fungal species, our data strongly indicated that *H. polymorpha* also possesses both forward and reverse transsulfuration pathways (Fig. 1D). Thus, like *S. cerevisiae, H. polymorpha* can grow in the presence of either methionine or cysteine as the sole sulfur source. However, a novel feature of *H. polymorpha* is the lack of direct incorporation of sulfide into homocysteine due to the absence of a *MET17* (or *MET25*) homolog. As a result, biosynthesis of methionine in *H. polymorpha* is possible only through cysteine and cystathionine, i.e. the forward transsulfuration reaction, which is mediated by *HpSTR2* and *HpSTR3* proteins. It is quite intriguing that sulfur metabolism of *H. polymorpha* is centered on cysteine rather than methionine, in contrast to other hemiascomycetes such as *S. cerevisiae* and *Kluyveromyces lactis* [30].

The comparative analysis of ^35^S-Met and ^35^S-Cys labeling experiments (Fig. 7, Figure S3) showed that the incorporation efficiency of cysteine into sulfur compounds was much higher than that of methionine in *H. polymorpha*, which is quite different from *S. cerevisiae*. The use of cysteine as preferential organic sulfur source partly reflects the cysteine-centered sulfur metabolism of *H. polymorpha*. Moreover, genome sequence analysis reveals that *H. polymorpha* possesses *HpYCT1*, a homolog of the *S. cerevisiae ScYCT1* encoding a high-affinity cysteine-specific transporter [31], but no homolog of the *S. cerevisiae MUP1* encoding a high-affinity methionine permease [32]. Furthermore, in contrast to *S. cerevisiae*, which possesses a family of multiple methionine transporter genes, *H. polymorpha* has only one ORF encoding methionine transporter, *HpMUP3*, which is a homolog of *S. cerevisiae MUP3* encoding a low-affinity methionine permease.

Our qRT-PCR data on transcript level analysis (Fig. 6) strongly suggest that the entire sulfur assimilation pathway leading to cysteine biosynthesis has evolved to be up-regulated upon sulfur limitation in *H. polymorpha*. Considering cysteine-centered sulfur metabolic pathway of *H. polymorpha* is quite unique among those of other yeast and filamentous fungal species, it will be intriguing to investigate the regulatory mechanisms of *H. polymorpha* sulfur pathway. Especially, the role of a *H. polymorpha* homolog of *S. cerevisiae* Met4p, a positive trans-acting sulfur regulatory protein related to the bZIP protein family [23], would be an intriguing subject for further study. While carrying out sequence analysis, we noticed that *H. polymorpha* Met4p is considerably smaller (330 amino acid residues) than ScMet4p (672 amino acid residues), reflecting different structural organization between two yeast Met4 homologs (Table S2). Therefore, molecular genetics and functional genomic studies on regulatory network mediated by HpMet4p would provide further insight into how this novel sulfur metabolic pathway is regulated in *H. polymorpha*, broadening our basic understanding of the conservation and divergence of the sulfur metabolic networks among eukaryotic organisms. On the other hand, from a biotechnological viewpoint, comprehensive knowledge of the sulfur pathway and its regulation might be usefully applied in the development of the artificial genetic circuit by fine-tuning of sulfur metabolism in yeast and filamentous fungal species to produce high-valued sulfur-containing amino acid and metabolites.

## Supporting Information

Figure S1 **Northern blot analysis of transcriptional regulation of** ***HpMET3*** **,** ***HpMET10*** **,** ***HpCYS1*** **, and** ***HpCYS3*** **.** Yeast cells were grown in YPD to the exponential phase and then transferred to B-medium. For Cd exposure, yeast cells grown in YPD to the exponential phase were transferred to YPD medium containing 0.6 mM Cd. After 2 hr cultivation, yeast cells were harvested and total RNA was extracted using the hot-phenol method. Total RNA was electrophoresed on 1.2% agarose-formaldehyde gels, blotted overnight onto a Nylon^+^ membrane, and hybridized with ^32^P-labeled DNA probes. DNA probes were labeled with the Rediprime II random priming labeling system kit (GE healthcare).
(TIF)

Figure S2 **Semi-quantitative reverse transcriptase-PCR (semiRT-PCR) analysis of transcriptional regulation of** ***H. polymorpha*** **genes in the sulfur pathway.** Expression of a selected set of genes was analyzed by semiRT-PCR. Yeast cells were grown in YPD to the exponential phase and then transferred to B-medium supplemented with the indicated 0.5 mM sulfur compounds. After 2 hr cultivation, yeast cells were harvested and total RNA was extracted for analysis.
(TIF)

Figure S3 **Analysis of incorporation efficiency of ^35^S-Cys or ^35^S-Met into** ***S. cerevisiae*** **and** ***H. polymorpha*** **cells.** Wild-type (WT) and *GSH1* null mutant (*gsh1*Δ) strains of *S. cerevisiae* (Sc) and *H. polymorpha* (Hp) were harvested after 2 hr labeling in the presence of 2 mM Cd. Total ^35^S-labeled compounds (20 µl) were extracted and quantified by liquid scintillation counting. The y-axis represents radioactivity measured as disintegrations per minute (dpm). Solid box, ^35^S-Cys-labeled samples; Empty box, ^35^S-Met-labeled samples.
(TIF)

Table S1 **Primers used for strain and plasmid construction.**
(DOCX)

Table S2 ***H. polymorpha*** **DL-1 genes involved in sulfur metabolism and regulation: GenBank accession numbers and identity to** ***S. cerevisiae*** **homologs.**
(DOCX)
