# Supplementary figures and images for: Novel Cysteine-Centered Sulfur Metabolic Pathway in the Thermotolerant Methylotrophic Yeast Hansenula polymorpha
Source: PLoS One. 2014 Jun 24;9(6):e100725. doi: 10.1371/journal.pone.0100725 (PMC4069077; doi:10.1371/journal.pone.0100725)

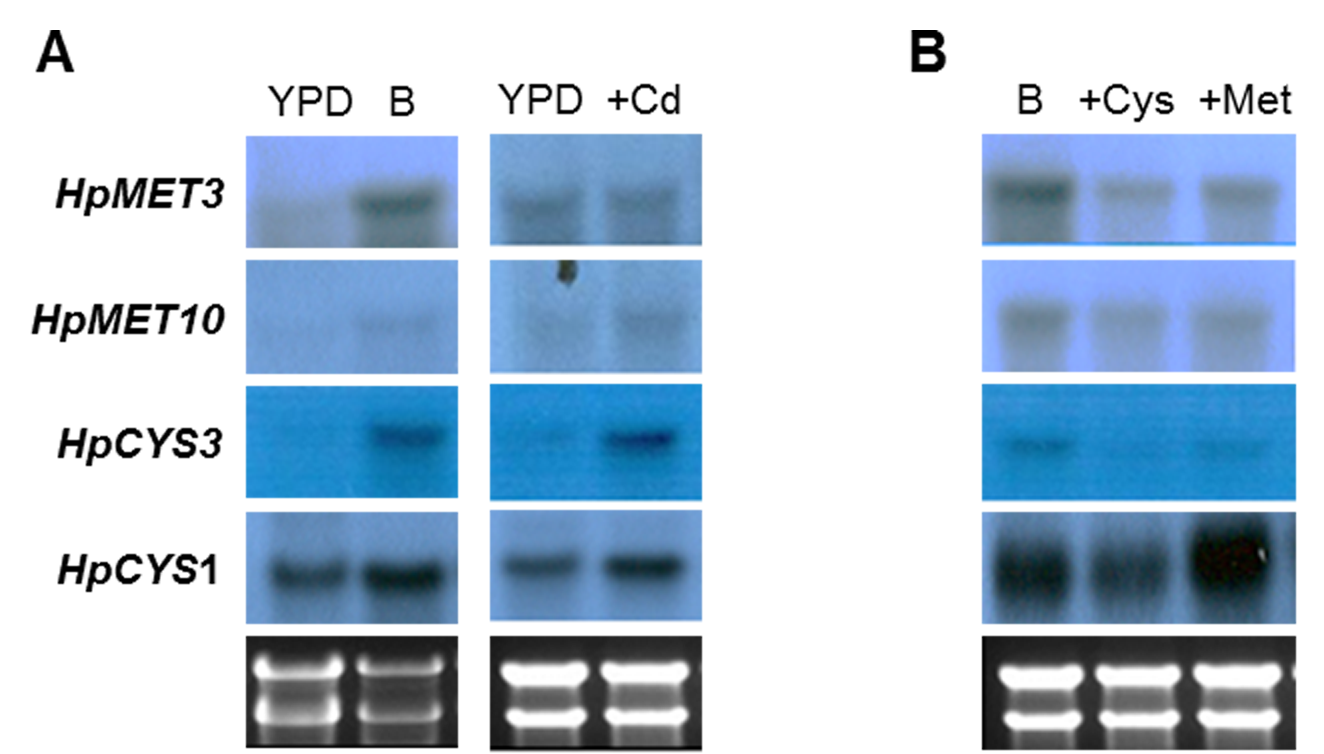

Supplement: Figure S1 — Northern blot analysis of transcriptional regulation of HpMET3 , HpMET10 , HpCYS1 , and HpCYS3 . Yeast cells were grown in YPD to the exponential phase and then transferred to B-medium. For Cd exposure, yeast cells grown in YPD to the exponential phase were transferred to YPD medium containing 0.6 mM Cd. After 2 hr cultivation, yeast cells were harvested and total RNA was extracted using the hot-phenol method. Total RNA was electrophoresed on 1.2% agarose-formaldehyde gels, blotted overnight onto a Nylon+ membrane, and hybridized with 32P-labeled DNA probes. DNA probes were labeled with the Rediprime II random priming labeling system kit (GE healthcare). (TIF) [file pone.0100725.s001.tif]

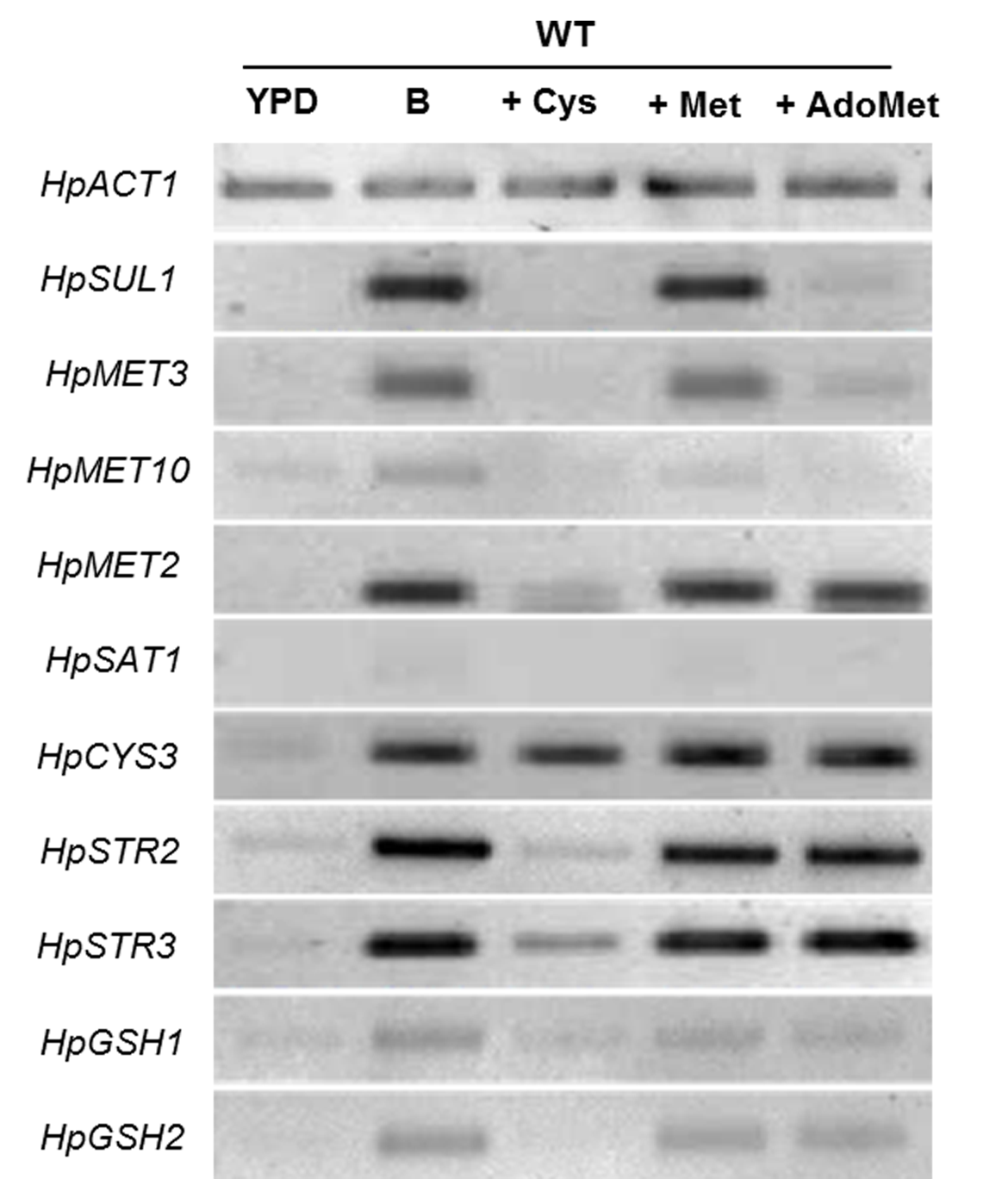

Supplement: Figure S2 — Semi-quantitative reverse transcriptase-PCR (semiRT-PCR) analysis of transcriptional regulation of H. polymorpha genes in the sulfur pathway. Expression of a selected set of genes was analyzed by semiRT-PCR. Yeast cells were grown in YPD to the exponential phase and then transferred to B-medium supplemented with the indicated 0.5 mM sulfur compounds. After 2 hr cultivation, yeast cells were harvested and total RNA was extracted for analysis. (TIF) [file pone.0100725.s002.tif]

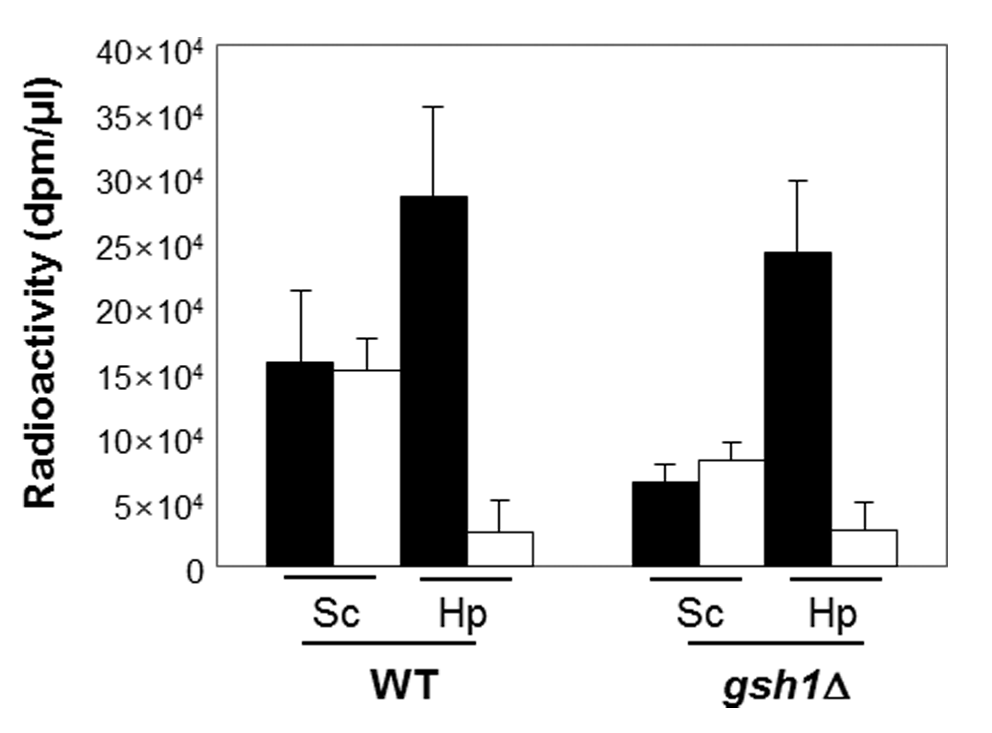

Supplement: Figure S3 — Analysis of incorporation efficiency of 35S-Cys or 35S-Met into S. cerevisiae and H. polymorpha cells. Wild-type (WT) and GSH1 null mutant (gsh1Δ) strains of S. cerevisiae (Sc) and H. polymorpha (Hp) were harvested after 2 hr labeling in the presence of 2 mM Cd. Total 35S-labeled compounds (20 µl) were extracted and quantified by liquid scintillation counting. The y-axis represents radioactivity measured as disintegrations per minute (dpm). Solid box, 35S-Cys-labeled samples; Empty box, 35S-Met-labeled samples. (TIF) [file pone.0100725.s003.tif]
