# Supplementary material for: Novel Cysteine-Centered Sulfur Metabolic Pathway in the Thermotolerant Methylotrophic Yeast Hansenula polymorpha
Source: PLoS One. 2014 Jun 24;9(6):e100725. doi: 10.1371/journal.pone.0100725 (PMC4069077; doi:10.1371/journal.pone.0100725)
Supplement: Table S1 — Primers used for strain and plasmid construction. (DOCX) [file pone.0100725.s004.docx]

Table S1. Primers used for strain and plasmid construction

| Name | Sequence | |  |
| --- | --- | --- | --- |
| **Gene deletion** | | |  |
| CYS1(NF) | 5’GCCGGCGACGTAAGAAGC 3’ | |  |
| CYS1(NR) | 5’AGCTCGGTACCCGGGGATCCGCTGCTAAATAGACGTTTG 3’ | |  |
| CYS1(CF) | 5’GCACATCCCCCTTTCGCCAGCTGAAGACTCAAAATCCAG 3’ | |  |
| CYS1(CR) | 5’CTAGACCAGTTCAAATCTAGC 3’ | |  |
| LacZ(NF) | 5’TCC CCG GGTACCGAGCT 3’ | |  |
| LacZ(NR) | 5’CACCGGTAGCTAATGATCCC 3’ | |  |
| LacZ(CF) | 5’CGAACATCCAAGTGGGCCGA 3’ | |  |
| LacZ(CR) | 5’CTGGCGAAAGGGGGATGTGC 3’ | |  |
| SATN-1F | 5’ GAGCGTTGTGTCATCTTCGG 3’ | |  |
| SATN-2B | 5’ AGCTCGGTACCCGGGGATCCACACCGCGAGAAGTGTCTTG 3’ | |  |
| SATN-3F | 5’ GCACATCCCCCTTTCGCCAGTCCTGTACATCTCCAAGGCG 3’ | |  |
| SATN-4F | 5’ GGTGTCGTGGCCGTAGAAG 3’ | |  |
| STR2(NF) | 5’GGAGGGACGAAAAAGCAGG 3’ | |  |
| STR2(NR) | 5’AGCTCGGTACCCGGGGATCCCCAACAGCCGTGAGAGC 3’ | |  |
| STR2(CF) | 5’GCACATCCCCCTTTCGCCAGGACTTCGAGGCGCGGTCC 3’ | |  |
| STR2(CR) | 5’ CCCAAGACACCCAAGATTC 3’ | |  |
| STR3(NF) | 5’ ATTGGAGTTGCAACAAAAGATGGGG 3’ | |  |
| STR3(NR) | 5’ AGCTCGGTACCCGGGGATCCGGCCTTGAAAGTTGCAG 3’ | |  |
| STR3(CF) | 5’ GCACATCCCCCTTTCGCCAGGGGTTGGAATCTCATCC 3’ | |  |
| STR3(CR) | 5’ GGTGTTCATAACAATCACGG 3’ | |  |
| CYS3(NF) | 5’ GGAACAGTTGGAGAAGCAC 3’ | |  |
| CYS3(NF) | 5’ AGCTCGGTACCCGGGGATCCCGCGAAGAACTCTGGGC 3’ | |  |
| CYS3(CF) | 5’ GCACATCCCCCTTTCGCCAGGAGATGTGCTTACCCTGG 3’ | |  |
| CYS3(CR) | 5’ GCAACTAGCTGACTTTTATTG 3’ | |  |
| CYS4a(NF) | 5’ CGTAGTCGTCTTCTGAGTCC 3’ | |  |
| CYS4a (NF) | 5’ AGCTCGGTACCCGGGGATCCGTCTAGAATACACAGGGTCC 3’ | |  |
| CYS4a (CF) | 5’ GCACATCCCCCTTTCGCCAGCCTTCCTCGGAACCACCA 3’ | |  |
| CYS4a (CR) | 5’ GGTCTGATTATGTAAGAGGC 3’ | |  |
| CYS4b(NF) | 5’ CCCAGATGGCATCCAATTTG 3’ | |  |
| CYS4b (NF) | 5’ AGCTCGGTACCCGGGGATCCGATAATTGCCAAAGCAAC 3’ | |  |
| CYS4b (CF) | 5’ GCACATCCCCCTTTCGCCAGGACAATCTTGGCGGACCC 3’ | |  |
| CYS4b (CR) | 5’ CCCCTCAGGTCTGAAGGGGC 3’ | |  |
| CYS4c(NF) | 5’ CGCGGATCCGCACTCTGCGTGCATCTG 3’ | |  |
| CYS4c (NF) | 5’ CGGAATTCGGGGCCTCGTAAAGATTC 3’ | |  |
| CYS4c (CF) | 5’ CGGAATTCTAGCGAGGTCAGCGCCAG 3’ | |  |
| CYS4c (CR) | 5’ CCCAAGCTTTTGAAGCCCAAAGTCTATTTG 3’ | |  |
| LacZ Eco(NF) | 5’ CGGAATTCATCCCCGGGTACCGAGCT 3’ | |  |
| LacZ Eco(CR) | 5’ GGAATTCCTGGCGAAAGGGGGATGTGC 3’ | |  |
|  |  | |  |
| **qRT-PCR analysis** |  | |  |
| HpACT1F-RT | 5’TCCAGGCTGTGCTGTCGTTG3’ | |  |
| HpACT1B-RT | 5’CCGGCCAAGTCGATTCTCAA3’ | |  |
| HpCYS3 qRT-PCR 1F | 5’GGAGCAGCTTGAAGGATCCG3’ | |  |
| HpCYS3 qRT-PCR 2B | 5’GAGCCCTGCTTGTCGGCAAT3’ | |  |
| HpSAT1 qRT-PCR 1F | 5’TTCTGTTCCCTGCGTGGCAG3’ | |  |
| HpSAT1 qRT-PCR 2B | 5’ATGTGGTCGAGCGCAAGCAG3’ | |  |
| HpMET3 qRT-PCR 1F-2 | 5’CGCTCAACATCTCCGGAACC3’ | |  |
| HpMET3 qRT-PCR 2B-2 | 5’CCCTGCAAGTACAGGGCGAA3’ | |  |
| HpMET2 qRT-PCR 1F-2 | 5’GATCTGCACCGTGCGTGATG3’ | |  |
| HpMET2 qRT-PCR 2B-2 | 5’TAGAGCGAGCATGCCTCCCA3’ | |  |
| HpSUL1 qRT-PCR 1F | 5’GGCTCTCATTGGCGGTGGAT3’ | |  |
| HpSUL1 qRT-PCR 2B | 5’TCGAGGTCGCGGTACTTTGG3’ | |  |
| HpSTR3 qRT-PCR 1F | 5’AGGAGACATGAACAGCGGCG3’ | |  |
| HpSTR3 qRT-PCR 2B | 5’GGTCGTCCTGGAATCCCCAA3’ | |  |
| HpMET10 qRT-PCR 1F | 5’TGACCGACCTCATGATGGCC3’ | |  |
| HpMET10 qRT-PCR 2B | 5’CTTCAACCTCCTTGGCGCTG3’ | |  |
| HpSTR2 qRT-PCR 1F | 5’TGGGACCAGGACGCGATCTA3’ | |  |
| HpSTR2 qRT-PCR 2B | 5’GACGGCATCGTCGACGGATA3’ | |  |
| HpGSH1 qRT-PCR 1F | 5’ACGGATCCGGCTCTTTCGAG3’ | |  |
| HpGSH1 qRT-PCR 2B | 5’TGCCGAGGCTCTGAAGCTCA3’ | |  |
| HpGSH2 qRT-PCR 1F-1 | 5’CGGATTTGCGGACGAGGAAC3’ | |  |
| HpGSH2 qRT-PCR 2B-1 | 5’GGGGCTTCAACACAAACCGC3’ | |  |
|  | |  | |
|  |  | |  |
