# Supplementary material for: Novel Cysteine-Centered Sulfur Metabolic Pathway in the Thermotolerant Methylotrophic Yeast Hansenula polymorpha
Source: PLoS One. 2014 Jun 24;9(6):e100725. doi: 10.1371/journal.pone.0100725 (PMC4069077; doi:10.1371/journal.pone.0100725)
Supplement: Table S2 — H. polymorpha DL-1 genes involved in sulfur metabolism and regulation: GenBank accession numbers and identity to S. cerevisiae homologs. (DOCX) [file pone.0100725.s005.docx]

Table S2. *H. polymorpha* DL-1 genes involved in sulfur metabolism and regulation: GenBank accession numbers and identity to *S. cerevisiae* homologs

| Accession number | Protein name | Gene | Identity (%) |
| --- | --- | --- | --- |
| JN676924 | Sulfate permease | *HpSUL1* | 51 |
| JN676925 | ATP sulfurylase | *HpMET3* | 62 |
| JN676926 | Adenylylsulfate kinase | *HpMET14* | 72 |
| JN676927 | 3'-phosphoadenylsulfate reductase | *HpMET16* | 62 |
| JN676928 | Sulfite reductase beta subunit | *HpMET5* | 52 |
| JN676929 | Subunit alpha of assimilatory sulfite reductase | *HpMET10* | 44 |
| JN676930 | Homoserine-O-acetyltransferase | *HpMET2* | 55 |
| JN676931 | Serine-O-acetyltransferase | *HpSAT1** | - |
| JN676932 | Cystathionine gamma-synthase | *HpSTR2* | 50 |
| JN676933 | Cystathionine beta-lyase | *HpSTR3* | 52 |
| JN676934 | Cysteine synthase | *HpCYS1** | - |
| JN676935 | Cystathionine gamma-lyase | *HpCYS3* | 12 |
| JN676937 | Cystathionine beta-synthase | *HpCYS4a* | 31 |
| JN676936 | Cystathionine beta-synthase | *HpCYS4b* | 21 |
| JN676938 | Cystathionine beta-synthase | *HpCYS4c* | 15 |
| JN676939 | Gamma glutamylcysteine synthetase | *HpGSH1* | 52 |
| JN676940 | Glutathione synthetase | *HpGSH2* | 50 |
| JN676941 | Glutathione *S*-transferase | *HpGTT1* | 22 |
| JN676942 | Glutaredoxin | *HpTTR1* | 25 |
| JN676943 | Glutathione-peroxidase | *HpHYR1* | 62 |
| JN676944 | Cobalamin-independent methionine synthase | *HpMET6* | 78 |
| JN676945 | *S*-adenosylmethionine sythetase | *HpSAM2* | 77 |
| JN676946 | *S*-adenosyl-L-homocysteine hydrolase | *HpSAH1* | 84 |
| KC840609 | bZIP transcription factor Met4p | *HpMET4* | 21 |

**H. polymorpha*-specific genes without *S. cerevisiae* homologs
